# Healthy eating in pregnancy, education for midwives: A pre-post intervention study

**DOI:** 10.18332/ejm/120004

**Published:** 2020-05-20

**Authors:** Shwikar M. E. Othman, Mary Steen, Julie-Anne Fleet, Rasika Jayasekara

**Affiliations:** 1 Obstetrics and Gynaecology Nursing Department, Faculty of Nursing, South Valley University, Luxor, Egypt; 2 UniSA Clinical and Health Sciences, University of South Australia, Adelaide, Australia

**Keywords:** knowledge, midwives, confidence, midwifery education, healthy eating education, diet and nutrition

## Abstract

**INTRODUCTION:**

Midwives have an important role in providing education in healthy eating to pregnant women, which is essential for maternal and foetal health and wellbeing. Importantly, midwives require continual professional development to ensure they provide up-to-date education.

**METHODS:**

A pre-post intervention study utilised a purpose-designed questionnaire to collect data at three time points. Forty-four midwives completed the pre education questionnaire, 29 of these midwives attended the education intervention (workshop/webinar) and completed the immediately after questionnaire. Nineteen midwives then completed a questionnaire at 6–8 weeks follow-up. The study aimed to evaluate midwives’ knowledge and level of confidence to discuss healthy eating in pregnancy.

**RESULTS:**

Education in healthy eating improved midwives’ knowledge and level of confidence, which were maintained for six to eight weeks. The mean difference of total scores on knowledge and confidence between pre and immediately after education questionnaires showed a statistically significant improvement in nutrition knowledge (4.93 ± 1.62 vs 7.55 ± 1.55; p<0.001) and confidence level (22.05 ± 6.87 vs 31.48 ± 7.47; p<0.001). In terms of the mode of education, there was a significant increase in total knowledge scores for midwives who attended a workshop compared to a webinar.

**CONCLUSIONS:**

Overall, healthy eating education improved midwives’ knowledge and confidence immediately after receiving education and also at 6–8 weeks follow-up. This study is unique as it evaluated midwives’ knowledge and level of confidence at 6–8 weeks post education. This study concludes that midwives benefited from receiving further knowledge on cultural food choices, eating behaviours, and dental care.

## INTRODUCTION

Midwives have an essential role to provide pregnant women with health and wellbeing education^1^. The midwives’ role includes the provision of healthy eating education to women in all maternity settings, including hospital, primary care and community centres^2,3^. However, there is evidence to suggest that midwives may not have received sufficient nutritional training to educate pregnant women, and have limited opportunities to attend nutrition-related continued professional development (CPD) sessions^2^.

A descriptive survey study conducted by Ilmonen et al.^4^ investigated nutrition knowledge and education needs of nurses and midwives who provide nutrition education. This study concluded that nursing and midwifery staff considered nutrition education to be essential, but several challenges were identified, such as counselling for health promotion and adopting evidence-based guidelines for medical conditions^4^. Solutions were suggested to improve knowledge on nutrition provided for midwives, and these included providing more educational materials and increasing opportunities by scheduling sufficient time to provide nutrition education as in-service education in health clinics^4^. Recent research suggests that health professionals, including midwives prefer web-based education and self-directed learning to overcome limited time; however, face-to-face education was positively associated with improved nutrition knowledge^2^. A significant gap in midwives’ knowledge and confidence to discuss healthy lifestyles and nutrition education with pregnant women has been identified^3,5^. Evidence has suggested that time and resources are limited for midwives to engage in these discussions with pregnant women^3,5^. Previous studies that assessed midwives knowledge and confidence to provide diet and nutrition education did not include any follow-up with participants after attending education to assess its effectiveness over time^6^. Therefore, this current study included a follow-up evaluation at 6–8 weeks after midwives attended a healthy eating workshop/webinar.

The purpose of this study was to investigate and explore midwives’ knowledge and level of confidence to support pregnant women to eat healthily, pre and post attending a workshop/webinar.

## METHODS

### Study design

A pre, immediate, and post study design with an education intervention (healthy eating education workshop/webinar) was used to evaluate midwives’ knowledge and level of confidence using a questionnaire at three time points. The post education assessment was at 6–8 weeks follow-up.

### Settings

Workshops were held at the University of South Australia and at the Women and Children’s Hospital, Adelaide. Participants accessed the online webinar through a study website.

### Recruitment and participants

Midwives who resided in South Australia were invited to participate in the study. The study was advertised through the Australian Nursing and Midwifery Federation, the Australian College of Midwives (South Australia branch), Healthy Development Adelaide Association, Women and Children’s Hospital Network, and social media. Newsletters and flyers were distributed to advertise workshops and webinar through metropolitan and rural hospitals within South Australia. Ethics approval was obtained from the Human Research Ethics Committee in January 2018 (ID 200150), data collection and intervention commenced in April 2018 and was completed in December 2018. Written consent was obtained from each participant. Ethical considerations, anonymity and confidentiality, voluntary participation and withdrawal at any time, were addressed.

### Sample size

Based on a power calculation using a single-factor, repeated measures design, a sample of five participants in each mode of education (workshop or webinar), measured at three time points, achieved a 91% power to detect differences among the mean knowledge and level of confidence, using a Geisser-Greenhouse Corrected F-Test at a 0.05 significance level. Based on previous studies^6^ we recruited a higher number (n=44) to account for loss at follow-up across the three time points. This higher number of participants ensured an adequate sample size for each mode of education (workshop or webinar) to enable a comparison between groups.

### Education intervention (workshop/webinar)

The workshop/webinar aimed to enhance midwives’ knowledge and confidence about healthy eating during pregnancy and promote healthy eating behaviour. The healthy eating education workshop/webinar content^7^ covered most recent evidence-based guidelines related to healthy eating and dietary requirements, portion sizes, eating myths, vegans and vegetarians eating, and cultural food choices. In addition, other information about eating habits and healthy behaviours, dental care, probiotics and prebiotics were included. Midwives had the option to attend a workshop or webinar. The workshop duration was two hours and was facilitated by the primary researcher (SO) and two co-facilitators (MS, JF). Alternatively, midwives were able to access the webinar recorded by a co-facilitator (MS) from the study site.

### Questionnaire development

A semi-structured questionnaire was developed utilising previously validated questions^3,8,9^. Information was included to address specific health priorities and guidelines related to nutrition during pregnancy^10-17^. Questions were based on findings from a systematic review for the effectiveness of providing healthy eating education for midwives^6^.

The questionnaire included five sections: 1) previous nutrition education; 2) assessment of midwives level of confidence, based on nine questions using 5-point Likert scale confidence from 1 for ‘not confident at all’ to 5 for ‘very confident’; 3) assessment of midwives’ knowledge, based on twelve multiple choice questions; 4) midwives’ sociodemographic characteristics, collected only at pre education; and 5) an invitation to participate in a follow-up. The questionnaire was hosted at Google forms and accessed through a dedicated study website, participants who attended workshops had the option to complete paper copies of the questionnaire.

### Validity and reliability

A pilot of the questionnaire was undertaken to ascertain the validity and reliability^18,19^. To test validity, a panel of five midwives, three academics and two clinically-based, were consulted to comment on the questionnaire and to give their feedback (content and face validity). The panel recommended rewording some questions for more clarity. Following the rewording, all five midwives agreed that the questionnaire was clear, easy to read and understand. To estimate the questionnaire reliability, seven midwives were invited to pilot the questionnaire at two different time points. No difference was observed between responses (Cronbach alpha coefficient was higher than 0.8), indicating reliable internal consistency and high accuracy of responses.

### Evaluation of healthy eating education (workshop/webinar)

Before undertaking the education, midwives were invited to complete an anonymous pre education questionnaire to record their baseline sociodemographics, knowledge and level of confidence. A second questionnaire was then completed immediately after attending the workshop/webinar. Finally, participants were reminded to complete a post education questionnaire 6–8 weeks after attending the education workshops or webinar.

### Data analysis

Data were entered into the Statistical Package for Social Sciences IBM (SPSS) version 25. Data related to midwives’ knowledge and level of confidence were analysed using Wilcoxon signed-rank test^20^. This test examined and compared differences in nutrition knowledge and confidence before, immediately after the workshop/webinar education, and 6–8 weeks later. Due to the challenges in matching midwives’ responses across the three time point questionnaires and loss at follow-up, a fixed-effect model^21^ was not used. The association between confidence and knowledge scores and sociodemographic characteristics was analysed using the Mann-Whitney and Spearman correlation coefficient tests^20^. Statistical difference was set at p<0.05. A biostatistician assisted with the statistical analysis.

## RESULTS

**Figure 1:**
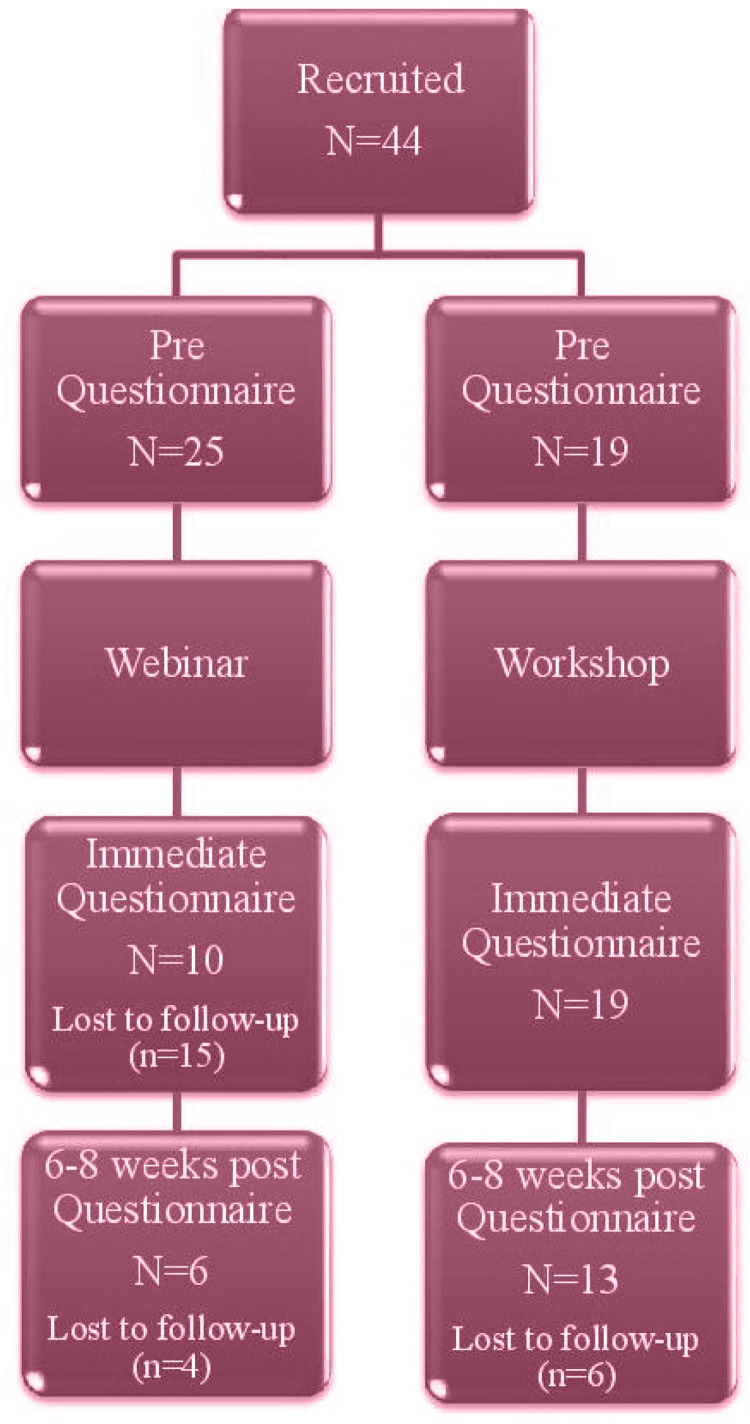
Recruitment flow chart (pre, immediate and post questionnaires) in 2018, South Australia

Forty-four midwives completed the pre education questionnaire, 29 of these midwives went on to attend either a workshop (n=19) or webinar (n=10). Nineteen midwives then completed the 6–8 weeks follow-up questionnaire (Figure 1).

### Sociodemographic characteristics

All forty-four participants who completed the pre education questionnaire were female, and the majority were over 41 years of age. The vast majority (n=41; 93.2%) practised midwifery in public hospitals, and 31 (70%) midwives had over ten years of midwifery experience. Nearly half (n=21; 47.7%) of the midwives reported that they had received nutrition education during undergraduate study or post qualification; only three midwives reported they had attended (CPD) nutrition education in the last 12 months, before attending the current education in healthy eating in pregnancy (Table 1). Mann-Whitney test was used to test the differences in midwives’ knowledge and level of confidence, between age, and years of midwifery experiences. This test showed no statistically significant differences between these variables.

**Table 1:** Sociodemographic characteristics of participant midwives in 2018, South Australia (N=44)

*Characteristics*	*n*	*%*
**Gender**		
Female	44	100.0
**Age** (years)		
20–30	5	11.4
31–40	7	15.9
41–50	12	27.3
>50	20	45.5
**Education level**		
Bachelor’s degree of midwifery	14	32.6
Hospital-based education midwifery	15	34.9
Midwifery postgraduate degree	13	30.2
Other qualification	13	30.2
**Midwifery experience** (years)		
<1	1	2.3
1–5	6	13.6
6–10	6	13.6
>10	31	70.5
**Principal place of practice**		
Public hospital	41	93.2
Private hospital	3	6.8
Independent/private midwifery practice	1	2.3
**Place of employment or work**		
Metropolitan public hospital	28	63.6
Metropolitan private hospital	3	6.8
Country hospital	11	25.0
Other	4	9.1
**Field of midwifery practice**		
a. Antenatal care	16	36.4
b. Labour	0	0.0
c. Postnatal	9	20.5
d. Rotation through all the above fields (a-c)	19	43.2
e. Group practice	3	6.8
f. Independent/private midwifery practice	1	2.3
g. Management	2	4.5
h. Education	13	29.5

### Midwives’ knowledge on healthy eating in pregnancy

Midwives’ knowledge was measured at all three time points (pre, immediate, and at 6–8 weeks post workshop/webinar). Midwives were asked to select the correct answers to 12 multiple-choice questions. Table 2 outlines the number of midwives who provided correct responses to knowledge questions at the three time points. Forty-four midwives initially completed the pre education questionnaire, and only 4 questions out of 12 were answered correctly by over 50% of participants. Questions that were not correctly answered related to daily energy requirement, vitamin D requirement, sources of omega-3, iodine requirements, folic acid requirement, daily water intake, calcium for vegans, and recommended weight gain (Table 2).

**Table 2:** Number of midwives who provided correct answers to assess their knowledge, based on pre, immediate, and post education questionnaires in 2018, South Australia

*Questions and correct answers*	*Pre (N=44)*		*Immediate (N=29)*		*Post (N=19)*					
	*n*	*(%)*	*n*	*(%)*	*n*	*(%)*
**International standard of recommended weight gain during a healthy pregnancy**						
11.3 –15.9 kg	21	(47.7)	18	(62.1)	12	(66.7)
**Approximate daily energy requirement for a healthy pregnant woman during the third trimester, as recommended by Australian guidelines**						
Additional 1900 kilojoules/day (454 kilocalories/day)	2	(4.5)	1	(3.4)	6	(31.3)
**Recommended daily water intake for pregnant women**						
Up to 3 litres (8 glasses) a day	18	(40.9)	23	(79.3)	15	(78.9)
**Recommended sources of fibre which pregnant women should eat daily**						
All of the above (cereals, plant dairy foods, fruit and vegetables)	23	(52.3)	13	(44.8)	9	(47.4)
**Appropriate sources of low glycaemic index GI carbohydrates for pregnant women**						
Chickpeas and cereals	39	(88.6)	28	(96.6)	19	(100.0)
**Most important vitamins for pregnant women who are vegetarians**						
Vitamins C, D and B12	26	(60.5)	27	(93.1)	18	(94.7)
**Recommended iodine requirement during pregnancy**						
150 μg/day	12	(27.3)	27	(93.1)	16	(84.2)
**Recommended sources of omega-3 during pregnancy**						
All of the above (vegetable oils, 2 servings of seafood per week, omega-3 fatty acid supplements)	11	(25.0)	17	(58.6)	7	(36.8)
**Approximate daily folic acid requirement for healthy pregnant woman as recommended by Australian guidelines**						
500 μg/day	17	(38.6)	17	(58.6)	11	(61.1)
**Recommended sources of calcium for vegan pregnant woman**						
Soybeans, soy yogurt and soy milk	20	(45.5)	15	(53.6)	14	(73.7)
**Recommended sources of protein during pregnancy**						
All of the above (lean meat, chicken and fish, dried beans, lentils and other legumes, milk, cheese, and yogurt	29	(65.9)	21	(72.4)	11	(57.9)
**Recommended vitamin D requirement during a healthy pregnancy**						
600 units/day	5	(11.4)	23	(79.3)	9	(47.4)

Immediately after attending the education workshop/ webinar, midwives’ responses improved as 10 out of 12 questions were answered correctly by over 50% of participants. At 6–8 weeks follow-up after the education, 8 out of 12 questions were answered correctly by over 50% of participants (Table 2). Answers to two questions relating to the approximate daily energy requirement and sources of fibre remained low at the immediate and 6–8 weeks evaluation time points, whereas answers for the two questions relating to vitamin D and omega-3 improved immediately after attending the healthy eating education, but the knowledge was not retained at the 6–8 weeks follow-up (Table 2).

A highly statistically significant difference in total knowledge scores was found between pre and immediately after education (immediate) questionnaires (p<0.001), and immediate with post education questionnaires (p<0.001). The range of correctly answered questions indicated an increase in midwives’ knowledge, which was maintained 6–8 weeks later (Table 3).

**Table 3:** Midwives’ total score on knowledge and level of confidence, based on pre, immediate, and post education questionnaires in 2018, South Australia

*Total score*	*PreN=44*	*ImmediateN=29*	*PostN=19*	*p^a^*	*p^b^*
**Knowledge**					
Mean ± SD	4.93 ± 1.62	7.55 ± 1.55	7.32 ± 1.53	0.000*	0.000*
Median (Range)	5.0 (2.0-8.0)	8.0 (4.0-10.0)	8.0 (5.0-10.0)		
**Confidence**					
Mean ± SD	22.05 ± 6.87	31.48 ± 7.47	30.89 ± 7.19	0.000*	0.000*
Median (Range)	21.0 (10.0-45.0)	32.0 (13.0-45.0)	32.0 (14.0-45.0)		

a Comparison between pre and immediately after education questionnaires.

b Comparison between immediately after and post education questionnaires.

* Wilcoxon signed-rank test, statistically significant difference (p<0.05).

### Midwives’ level of confidence in educating on healthy eating in pregnancy

Nine questions related to the midwives’ level of confidence in providing healthy eating education. The majority of midwives who completed the pre, immediate and post education questionnaires answered question to indicate they were ‘confident’ or ‘moderately confident’ to provide general nutrition education (Table 4). Midwives perceived their role to provide pregnant women with nutrition advice as an integral part of their midwifery practice. However, more than half of the midwives reported that they were ‘not confident at all’ to ‘slightly confident’ to provide education for vegetarians, vegans and cultural choices and ethnic minorities before the education. This improved for midwives who attended the education, ranging between ‘confident’ and ‘moderately confident’, and responses remained the same for midwives who completed the questionnaire at 6–8 weeks follow-up (Table 4).

**Table 4:** Midwives’ level of confidence to provide healthy eating education, based on pre, immediate, and post education questionnaires in 2018, South Australia

*Confidence*	*Pre (N=44)*		*Immediate (N=29)*		*Post (N=19)*	
	*n*	*(%)*	*n*	*(%)*	*n*	*(%)*
**To provide general nutrition education to pregnant women**						
Not confident at all	1	(2.3)	0	(0.0)	0	(0.0)
Slightly confident	4	(9.1)	1	(3.4)	0	(0.0)
Confident	19	(43.2)	7	(24.1)	4	(21.1)
Moderately confident	18	(40.9)	17	(58.6)	12	(63.2)
Very confident	2	(4.5)	4	(13.8)	3	(15.8)
**To dispel any eating myths about what and how much to eat during pregnancy**						
Not confident at all	10	(23.3)	2	(6.9)	0	(0.0)
Slightly confident	11	(25.6)	1	(3.4)	0	(0.0)
Confident	9	(20.9)	4	(13.8)	3	(16.7)
Moderately confident	11	(25.6)	13	(44.8)	10	(55.6)
**To give health education for vegetarians**						
Not confident at all	15	(34.1)	0	(0.0)	0	(0.0)
Slightly confident	9	(20.5)	6	(20.7)	2	(10.5)
Confident	14	(31.8)	4	(13.8)	5	(26.3)
Moderately confident	4	(9.1)	14	(48.3)	10	(52.6)
Very confident	2	(4.5)	5	(17.2)	2	(10.5)
**To give health education for vegans**						
Not confident at all	9	(20.5)	1	(3.4)	1	(5.3)
Slightly confident	14	(31.8)	3	(10.3)	2	(10.5)
Confident	12	(27.3)	7	(24.1)	8	(42.1)
Moderately confident	6	(13.6)	15	(51.7)	6	(31.6)
Very confident	3	(6.8)	3	(10.3)	2	(10.5)
**To give health education according to cultural choices**						
Not confident at all	13	(29.5)	2	(6.9)	1	(5.3)
Slightly confident	18	(40.9)	3	(10.3)	3	(15.8)
Confident	10	(22.7)	10	(34.5)	8	(42.1)
Moderately confident	2	(4.5)	12	(41.4)	5	(26.3)
Very confident	1	(2.3)	2	(6.9)	2	(10.5)
**To provide dental care health education for pregnant women**						
Not confident at all	12	(27.3)	1	(3.4)	0	(0.0)
Slightly confident	12	(27.3)	2	(6.9)	0	(0.0)
Confident	11	(25.0)	4	(13.8)	7	(36.8)
Moderately confident	7	(15.9)	13	(44.8)	7	(36.8)
Very confident	2	(4.5)	9	(31.0)	5	(26.3)
**To recommend herbal drinks in pregnancy**						
Not confident at all	10	(22.7)	2	(6.9)	1	(5.3)
Slightly confident	19	(43.2)	5	(17.2)	4	(21.1)
Confident	11	(25.0)	12	(41.4)	9	(47.4)
Moderately confident	3	(6.8)	7	(24.1)	2	(10.5)
Very confident	1	(2.3)	3	(10.3)	3	(15.8)
**To recommend diet for people from ethnic or minority groups**						
Not confident at all	8	(18.2)	2	(6.9)	1	(5.3)
Slightly confident	14	(31.8)	3	(10.3)	5	(26.3)
Confident	12	(27.3)	12	(41.4)	7	(36.8)
Moderately confident	7	(15.9)	8	(27.6)	4	(21.1)
Very confident	3	(6.8)	4	(13.8)	2	(10.5)
**To recommend diet for people with previous or complex medical conditions**						
Not confident at all	16	(36.4)	3	(10.3)	3	(15.8)
Slightly confident	19	(43.2)	7	(24.1)	3	(15.8)
Confident	7	(15.9)	10	(34.5)	9	(47.4)
Moderately confident	1	(2.3)	8	(27.6)	2	(10.5)
Very confident	1	(2.3)	1	(3.4)	2	(10.5)

A statistically significant difference for midwives’ total confidence score was found between pre and immediate education questionnaires, as well as the immediate and post education questionnaires (Table 3).

### Association between midwives’ total knowledge and confidence score

A positive correlation was found between midwives’ knowledge score and confidence score over the three time point questionnaires, with a highly statistically significant difference (p<0.007) at the pre education evaluation (Supplementary file, Table S1).

In terms of the mode of education, responses from midwives who attended a workshop had significantly higher knowledge scores, both immediately after education and at the 6–8 weeks post education follow-up, compared to midwives who completed the webinar (Table 5).

**Table 5:** Comparison between mode of education and its effect on knowledge and level of confidence, based on pre, immediate, and post education questionnaires in 2018, South Australia

		*Mode of education*		*P*
		*Workshop*	*Webinar*	
**Pre education** (N=44)	**Confidence score**				0.440
	Mean ± SD	22.42 ± 6.53	21.76 ± 7.24		
	Median (Range)	22.0 (10.0-34.0)	21.0 (11.0-45.0)		
	**Knowledge score**				0.029*
	Mean ± SD	5.47 ± 1.50	4.52 ± 1.61		
	Median (Range)	6.0 (2.0-8.0)	4.0 (2.0-8.0)		
**Immediately after education** (N=29)	**Confidence score**				0.872
	Mean ± SD	31.42 ± 7.73	31.60 ± 7.37		
	Median (Range)	32.0 (13.0-45.0)	31.5 (19.0-40.0)		
	**Knowledge score**				0.031*
	Mean ± SD	8.05 ± 1.27	6.60 ± 1.65		
	Median (Range)	8.0 (6.0-10.0)	6.5 (4.0-9.0)		
**Post education** (N=19)	**Confidence score**				0.332
	Mean ± SD	31.85 ± 5.61	28.83 ± 10.17		
	Median (Range)	32.0 (23.0-44.0)	29.0 (14.0-45.0)		
	**Knowledge score**				0.043*
	Mean ± SD	7.85 ± 1.28	6.17 ± 1.47		
	Median (Range)	8.0 (5.0-10.0)	5.5 (5.0-8.0)		

* Statistically significant difference (p<0.05).

## DISCUSSION

The purpose of this study was to assess midwives’ knowledge and level of confidence before, immediately after and at 6–8 weeks follow-up after attending healthy eating in pregnancy workshop or webinar. This study demonstrated that for midwives who completed the follow-up questionnaire, this healthy eating education was effective in increasing their knowledge and level of confidence. Overall, before attending the workshop/webinar, midwives had a low level of confidence when answering questions related to vegans, dental care and cultural choices. For midwives who received education, their responses indicate that there was an improvement in their knowledge of healthy eating and nutrition. In particular, their knowledge regarding water intake, sources of low Glycaemic Index (GI), iodine, and essential vitamins for vegetarian pregnant women was shown to have increased. This is evidenced by an increase in correct responses immediately after education and at the 6–8 weeks follow-up.

### Midwives’ knowledge before attending the healthy eating education workshop/webinar

Prior to attending the workshop/webinar, a range of responses to each question indicated that some midwives had insufficient knowledge of recent Australian guidelines for nutrition and diet. Interestingly, in our study, a large proportion of midwives gave incorrect responses to international standards of recommended weight gain for a healthy pregnancy, which is consistent with other studies. A previous study reported that a sample from 386 midwives (n=180; 46.6%) provided incorrect responses^8^, and another similar study reported a low level of knowledge for recommended weight gain (n=241;

73.3%)^3^. A possible explanation for this deficit in knowledge maybe that Australia adheres to the Institute of Medicine recommendations, and midwives appeared to be unaware of these recommendations.

Daily energy requirements in pregnancy are essential for energy deposition in maternal and foetal tissues^22^. Recommended daily intake for energy requirements vary between countries; however, it is agreed that additional requirements are relatively small. The current study demonstrated a deficit in midwives’ knowledge, similarly a previous study^3^ reported that midwives did not appear to have up-to-date knowledge about Australian guidelines on daily energy requirements during the third trimester^17^.

Before the workshop/webinar, midwives demonstrated an adequate knowledge regarding food sources of low GI, protein, and vitamins, especially vitamins C, D and B12, which are essential for pregnant women who are vegetarians^23^. Similarly, Arish et al.^3^ reported that 205 (62%) of midwives gave correct answers for essential vitamins for vegetarian pregnant women. Furthermore, the current study highlighted that the majority of midwives did not respond correctly to fluid intake recommendations during pregnancy; while national guidelines recommend that pregnant women need to increase their fluid intake to about 3 L or up to 6–8 glasses/day^24^. Pregnant women should also consume an adequate amount of fibre daily to prevent minor discomfort^25^. However, in the current study, responses indicate that some midwives need to improve their knowledge of alternative sources of fibre.

Iodine is essential for healthy brain development and the nervous system in a developing baby and young infant, which increases a woman’s need for iodine requirements during pregnancy^26^. It is recommended that a pregnant woman should take 150 μg/day of iodine^27^. Arrish et al.^3^ identified a deficit in Australian midwives’ knowledge about iodine requirements, which was also demonstrated in this current study by incorrect answers before education. Similarly, a study was undertaken in the UK involving 60 midwives and reported that 67% did not discuss iodine requirements while providing education to pregnant women, and a vast majority (95%) indicated a need for further education^14^. In this current study, a large proportion of midwives were over 50 years of age and did not report opportunities in undertaking CPD education related to nutrition during undergraduate or post-qualifying; therefore, this also indicates that there is a need for further education.

Recently, vitamin D recommendations during pregnancy have been a controversial issue, and two systematic reviews concluded that most trials investigating vitamin D during pregnancy were small and of low quality^28,29^. Therefore, the evidence is limited to guide clinical practice and policy recommendations, and this may have contributed to midwives’ poor correct response rate in the current study, as more than half of midwives gave incorrect answers for vitamin D recommendations, which indicates that midwives need to be updated on the recent guidelines. In addition, the current study highlighted that midwives have limited knowledge on recommended sources of omega-3 during pregnancy. However, health professionals need to consider and recommend to pregnant women the richest sources of omega-3 from seafood, although other sources include flaxseed oil and vegetable oils^30^.

Awareness about the importance of folic acid and recommendations during preconception and pregnancy have increased over the last few decades; however, it has been reported that pregnant women and some midwives still lack knowledge^31^, consistent with our findings. Interestingly, there is no internationally agreed standard for folic acid requirements as recommendations differ between countries. For example, in Australia, the recommended daily intake of folic acid is 500 μg/day^32^, whereas the international recommendation is 400 μg/day for healthy pregnant women starting at least one month before conception and continuing to 12 weeks of pregnancy^33^. The high proportion of midwives practising in Australia that have completed midwifery education and practice in other countries may partly explain why some midwives responded incorrectly.

This may also have implications as different countries have different laws on fortified foods with folates, such as cereal and fruit drinks.

The education in the current study was provided by either a workshop or webinar. Midwives who attended the workshop were more likely to demonstrate a significant increase in their knowledge than those who participated in the webinar. Interestingly, a systematic review identified nutrition education programs delivered face-to-face or by self-directed learning packs were associated with an improvement in midwives’ nutrition knowledge^2^. Improvement may be due to opportunities for discussion and also being able to ask questions and clarify any misunderstandings relating to the materials and resources, thus providing a two-way interaction between facilitator and participants leading to a positive impact on learning^2^. Whereas, an integrative review concluded that blended learning, combining online and face-to-face components, is suggested as the best way forward to provide the high levels of interactivity, reflection, practice and application to practice^34^.

### Midwives’ level of confidence before attending the education

Midwives level of confidence to discuss general and specific nutrition issues varied before attending the education, as responses indicated they felt confident to discuss general advice but were less confident to provide specific nutrition education (ranging from slightly confident to moderately confident). This finding is consistent with a UK study^14^ that involved 60 midwives who reported that they were very confident to discuss general nutrition knowledge regarding dietary guidelines; however, they were not confident to discuss requirements of iodine during pregnancy. In contrast, an Australian study^3^ reported that midwives level of confidence to discuss general nutrition advice and provide education was moderate; however, they were not confident to discuss specific diets. During the workshop/ webinar, midwives reported the importance of providing nutrition education for pregnant women who are vegans or vegetarians and discussed different food sources during the workshop/webinar. However, midwives also reported the need for further education and requested more information about cultural foods and eating behaviours.

Interestingly, midwives’ confidence to discuss eating behaviours with pregnant women, such as what and how much should women eat during pregnancy, ranged from ‘slightly confident’ to ‘moderately confident’. Similarly, a previous study reported that midwives’ confidence to address weight-related behaviours was low^35^, and midwives level of confidence to discuss weight-related risks and provide advice on practical strategies to achieve a good lifestyle were moderate^36^.

### Knowledge and level of confidence after attending the education

It was anticipated that midwives’ knowledge and level of confidence would improve immediately after education, and this was confirmed by the increase in correct answers. Moreover, midwives self-reported an improvement in their confidence across a range of questions. These findings are consistent with other studies included and reported in a systematic review^6^. No previous studies that have examined healthy eating education for midwives have reported a follow-up period after the education intervention^6^. Therefore, this current study is unique as it re-assessed the midwives’ knowledge and level of confidence after attending education at the 6–8 weeks follow up. Overall, findings demonstrate that knowledge and level of confidence were maintained over time for midwives who completed the post education questionnaire. This may be due to midwives showing an interest and revisiting workshop/webinar education materials during the 6–8 weeks follow-up period and/or taking time to research answers when prompted to complete their post education questionnaire.

### Strengths and limitations

A strength of this study was in providing education that was designed based on up-to-date international and Australian nutrition evidence-based guidelines. Another strength is, as far as the authors are aware, that this current study is the first to report on a more extended follow-up period after the education intervention. Findings suggest midwives’ knowledge and level of confidence improved and maintained over time for those who completed the followup questionnaire.

This study, however, does have limitations. While the response rate of midwives who completed the education workshop/webinar was low, the sample size is appropriate for the power calculation. Unfortunately, 15 midwives who completed the pre education questionnaire did not respond to email reminders to attend the workshop or webinar, and therefore did not continue to participate in the study. In addition, a further ten midwives did not complete the questionnaire at the 6–8 weeks follow-up. The reason why midwives did not continue to participate in this study is not clear but it might have been be a heavy workload, lack of motivation and/or time constraints. Therefore, the risk of motivational bias needs to be considered, and the possibility that midwives who attended the workshop or webinar and participated in the full evaluation of this study were either highly motivated and interested in healthy eating education or were aware that they lacked knowledge or confidence and completed the education to address this deficit.

Providing healthy eating education based on evidencebased guidelines is recommended for continual professional development to improve and maintain midwives’ knowledge and level of confidence over time. Further research is needed to explore the best way of providing healthy eating education for midwives to enable them to increase and maintain knowledge and confidence. There is limited evidence to confirm or refuse if knowledge and confidence are maintained after 8 weeks following the healthy eating education. Therefore, longitudinal studies to assess the effectiveness of healthy eating education for midwives and how this may affect their clinical practice is urgently required.

## CONCLUSIONS

Based on this study findings, midwives who attended the healthy eating workshop/webinar overall improved their knowledge and levels of confidence immediately after the education and at 6–8 weeks follow-up. In particular, midwives demonstrated increased nutrition knowledge in water intake, sources of low GI, iodine, and in particular vitamin requirements for vegetarians. However, some midwives did not maintain their level of knowledge for specific nutrition topics, such as omega-3 and vitamin D requirements.

This study is novel in that it evaluated midwives’ knowledge and levels of confidence at 6–8 weeks after attending healthy eating education. This study concludes that midwives benefited from receiving this type of education but also that they specifically require further education on cultural food choices, eating behaviours and dental care.
